# Structural, Optical, and Vibrational Properties of Cerium Tungstate–Titanate Nanocomposite for Tetracycline Degradation

**DOI:** 10.1021/acsomega.6c01535

**Published:** 2026-05-21

**Authors:** Emerson da Silva do Nascimento, Suziete Batista Soares Gusmão, José Ferreira da Silva Júnior, Antonio Werbeson Miranda, Francisco das Chagas Silva Santos, Rodrigo Prado Feitosa, Tainara Gomes de Oliveira, Alexandre Silva Santos, Thiago de Lourenço e Vasconcelos, Mônica Rodrigues de Sá, Eduardo Padrón-Hernández, Bartolomeu Cruz Viana, Gustavo Oliveira de Meira Gusmão

**Affiliations:** † Graduate Program in Physics, 67823 Federal University of Piauí, Teresina, Piauí CEP 64049-550, Brazil; ‡ Codó Science Center, Federal University of Maranhão, Codó, Maranhão CEP 65400-000, Brazil; § Graduate Program in Materials Science and Engineering, 67823 Federal University of Piauí, Teresina, Piauí CEP 64049-550, Brazil; ∥ Federal Institute of Piauí, Campus Oeiras, Oeiras do Piauí, Piauí CEP 64500-000, Brazil; ⊥ Department of Analytical Chemistry, Faculty of Pharmacy, University of Sevilla, Sevilla ES 41012, Spain; # Optical Spectroscopy Laboratory, Instituto de Física, 28127 Universidade de Brasília, Brasília-DF 70910-900, Brazil; ∇ Instituto Nacional de Metrologia, Qualidade e Tecnologia (INMETRO), Duque de Caxias, Rio de Janeiro 25250-020, Brazil; ○ Department of Physics, University of Pernambuco, Av. Prof. Luiz Freire s/n, Recife-PE CEP 50740-540, Brazil; ◆ Center for Open Distance Education - CEAD, Federal University of Piauí, Teresina CEP 64049-550, Brazil; ¶ Department of Physics, State University of Piauí, Teresina, Piauí CEP 64002-150, Brazil

## Abstract

In this study, we report on the structural, electronic, and vibrational properties of the sodium titanate nanotubes (NaTiNTs) modified with cerium tungstate (CeW), which were synthesized via a single-step hydrothermal procedure, resulting in the nanocomposite (TiNTs@CeW). Structural and spectroscopic characterizations confirmed the preservation of the nanotube morphology after modification, while XPS and STEM analyses indicated the deposition of Ce- and W-containing species on the nanotube surface. Optical properties were investigated by UV–vis absorption spectroscopy and photoluminescence. For the TiNTs@CeW sample, a significant reduction in the band gap from (3.23 ± 0.04) eV to (2.52 ± 0.03) eV was observed, as well as an increase in the Urbach energy from (0.316 ± 0.003) eV to (0.444 ± 0.001) eV, compared to NaTiNTs. These effects are associated with a higher concentration of oxygen holes and localized electronic states, which act as emission centers and temporary charge traps. The TiNTs@CeW showed significantly superior photocatalytic performance, promoting 73.84% degradation of tetracycline in high-concentration solution and exhibiting good stability and reusability. NaTiNTs achieved only 26.28% degradation under the same experimental conditions. These results highlight the potential of nanocomposite TiNts@CeW as effective materials in environmental remediation through their good photocatalytic performance in the degradation of the drug tetracycline.

## Graphical abstract

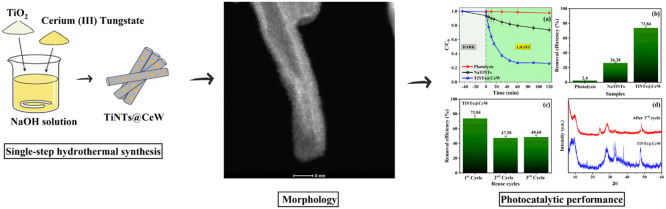

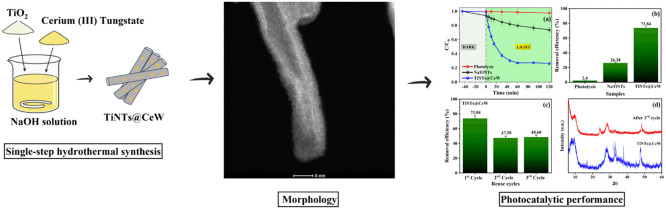

## 1 Introduction

The contamination of effluents and water bodies by improper disposal of organic pollutants, such as pharmaceuticals and dyes, is one of the main challenges in wastewater treatment.^1^ Conventional methods, including coagulation, sedimentation, and biological treatments, are generally ineffective in completely removing recalcitrant compounds, which exhibit high chemical stability and low biodegradability.^2^ This limitation can cause serious environmental impacts, compromising water quality, aquatic life, and human health. In this context, heterogeneous photocatalysis has emerged as a promising alternative, as it employs nanomaterials activated by UV or visible radiation to generate reactive oxygen species capable of degrading persistent organic pollutants, including pharmaceutical residues. In addition to its high efficiency, this process is versatile and environmentally sustainable.^3^

Pharmaceuticals, particularly antibiotics, have emerged as one of the most concerning contaminants, posing an increasing threat to the aquatic environment. Tetracycline, for example, widely used in human and animal medicine, exhibits activity against a wide range of microorganisms, including Gram-positive and Gram-negative bacteria.^4^ Owing to its proven efficacy in treating trachoma, pneumonia, and acute diarrhea, it has become a reliable and indispensable therapeutic agent in infection control and is widely adopted by healthcare professionals.^5,6^ However, owing to its low metabolic retention, approximately 70% of this antibiotic is released into the environment, predominantly via wastewater effluents.^6^ Additionally, TC is recognized for its greater environmental persistence, greater stability in aquatic systems, and greater resistance to biodegradation. These characteristics make tetracycline a more challenging and environmentally relevant model pollutant for evaluating photocatalytic performance under realistic conditions.^5–7^

Therefore, TiO_2_-based nanostructures, such as nanotubes, nanoparticles, and titanate nanorods, have attracted considerable attention from the scientific community in the field of heterogeneous photocatalysis due to their numerous advantages, including high porosity, large specific surface area, and excellent electron transfer mobility.^8^ Among these nanostructures, titanate nanotubes have been extensively studied owing to their layered nanotubular architecture, highly hydroxylated surface, and ion-exchange capacity, which enhance the properties of TiO_2_, such as bandgap reduction through ion insertion into their lamellar structure.^8–11^

In this context, the incorporation of ions or functional compounds into sodium titanate nanotubes has attracted increasing interest in tailoring their structural and electronic properties. Among these compounds, cerium tungstate, which crystallizes in a monoclinic structure belonging to the *C*2/*c* space group (no. 15), is well known for its luminescent properties.^12^ When derived from the scheelite structure, these compounds exhibit potential for a wide range of applications, including lasers,^13^ scintillators,^14^ catalysts,^15^ gas separation membranes,^16^ supercapacitors,^17^ and optical fibers,^18^ making them excellent modifiers for titanate nanotubes. According to Cruz et al.,^6^ tungsten-based materials with a tetragonal scheelite-type structure, or similar structures, show great potential for mitigating environmental pollution caused by antibiotics. Additionally, Turkay et al.^19^ demonstrated that cerium tungstate-based catalysts increase the concentration of oxygen vacancies and intensify redox cycles, favoring charge activation and pollutant degradation reactions.

However, studies that comprehensively investigate the structural, optical, and vibrational properties related to drug degradation in cerium tungstate and sodium titanate nanocomposites are scarce. Works such as that of Xiao et al.^20^ focus on WO_3_/titanate nanocomposites, in which WO_3_ is identified as a distinct crystalline phase anchored to the nanotubes, focusing on semiconductor–semiconductor coupling to improve photocatalysis. In contrast, CeW/sodium titanate systems predominantly involve interfacial interactions and surface modification, associated with defects and oxygen vacancies, whose influence on drug degradation has still not been well explored. This gap in research is mainly due to the difficulty of developing a synthesis route that allows this type of modification without compromising the nanotube morphology, in addition to the need to understand how the insertion of these nanoparticles can influence the structural, vibrational, and electronic properties of the resulting materials. Therefore, this study aimed to investigate the synthesis of cerium tungstate-modified sodium titanate nanotubes (TiNTs@CeW) and evaluate their structural, morphological, optical, and vibrational properties for tetracycline degradation applications.

## 2 Experimental Procedures

### 2.1 Materials

All reagents used were of above 98% analytical grade and did not require further purification. The reagents were as follows: Titanium Dioxide (TiO_2_-anatase phase, Sigma-Aldrich, 99% purity), Sodium Hydroxide (NaOH, Dinâmica, Indaiatuba, Brazil, 98% purity), Cerium­(III) Tungstate (Sigma-Aldrich, 99% purity, Product No: 520128), and Tetracycline (C_22_H_24_N_2_O_8_, Sigma-Aldrich, 98.0–102.0% - HPLC). All solutions were prepared using deionized water.

### 2.2 Synthesis

The synthesis of TiNTs@CeW was performed using an alkaline hydrothermal method adapted from.^21^ Initially, 0.12 g of TiO_2_ and 0.04 g of Cerium­(III) Tungstate were suspended in 12 mL of an aqueous NaOH solution and magnetically stirred for 10 h, resulting in a yellowish solution. The suspension was transferred to a Teflon cup and placed in a stainless-steel autoclave, filling 60% of the total volume. It was subjected to 150 °C for 72 h in an oven. The yellow solid was separated from the supernatant by centrifugation at 3500 rpm for 5 min and repeatedly washed with deionized water to remove unreacted residual species until the supernatant reached pH 11–12. Once the supernatant reached this pH, the liquid phase was separated from the solid phase, and the solid was subsequently dried under vacuum in a desiccator for 24 h. The material was ultimately obtained and received the name TiNTs@CeW.

### 2.3 Characterizations

The structural analysis of the samples was performed by X-ray diffraction (XRD) using CuKα radiation (λ = 1.5418 Å) on a Bruker D8 Advance diffractometer operating at 40 kV and 40 mA, with a scan rate of 2° min^–1^ over a 2θ range of 5–70°. The elemental composition of the NaTiNTs and TiNTs@CeW samples was analyzed using energy-dispersive spectroscopy (EDS). Semiquantitative analyses were conducted with the FEI microscope, model QUANTA FEG 250, operating at an acceleration voltage of 20 kV. High-resolution transmission electron microscopy (HRTEM) and scanning transmission electron microscopy (STEM) images of the samples were acquired using an FEI Titan 80–300 microscope, operated at an acceleration voltage of 200 kV.

The XPS analyses were performed on a Thermo Scientific K-Alpha instrument using Al Kα radiation as the source. Measurements for both samples were obtained with a spot size of 400 μm, standard lens mode, and CAE analysis mode, using a pass energy of 200.0 eV. The samples were mounted on a metal support with carbon tape and analyzed under high-vacuum conditions.

The average particle size, ζ-potential, and polydispersity index (PDI) were determined by dynamic light scattering (DLS) using a Zetasizer Nano ZS90 (Malvern Instruments, model DTS 1070), equipped with a polycarbonate cuvette containing beryllium/copper-coated gold electrodes. Before the measurements, the dispersions were subjected to 30 min of ultrasonic treatment, in which 1 mL of each sample was transferred to folded ζ-capillary cells.

Raman spectroscopy and photoluminescence (PL) analyses of the nanostructures were performed on the Horiba LabRam Evolution spectrometer equipped with a CCD system, using a 532 nm laser for Raman measurement and a 405 nm laser for PL as the excitation source, with an output power of 10 mW, 10 accumulations of 10 s, and a 50x lens. All Raman and photoluminescence (PL) measurements were performed at pressure and room temperature. Fourier transform infrared spectroscopy (FTIR) was performed using an attenuated total reflectance (ATR) mode setup on a Bruker Vertex 70 spectrometer. A total of 128 scans were conducted at a resolution of 2 cm^–1^ to obtain spectra with high signal-to-noise ratios. The spectral range utilized was 4000–400 cm^–1^. The study of the optical properties of the samples was carried out through diffuse reflectance spectroscopy (UV–vis) using a Shimadzu 2700 spectrometer.

### 2.4 Photocatalytic Application

The Tetracycline (TC) photodegradation test was conducted based on a previously described methodology with specific adaptations.^22^ This antibiotic was used as a model contaminant at a concentration of 40 ppm in 200 mL of aqueous solution. The concentration of the synthesized materials was adjusted to 0.10 g L^–1^.

Before irradiation, the samples were stirred in the dark for 45 min to ensure adsorption–desorption equilibrium. The photodegradation experiments were carried out under UV light using a 120 W lamp with an approximate light intensity of 3.2 klx for a maximum period of 120 min. Aliquots of 2 mL were collected at predetermined time intervals (0, 5, 10, 15, 30, 45, 60, 90, and 120 min) using a micropipette. The collected aliquots were analyzed by spectroscopy using a UV–vis spectrophotometer (Agilent Technologies Cary 60). To ensure complete separation of the catalyst, the samples were centrifuged before analysis. In the material reuse phase, after the end of each cycle, the solution was centrifuged to separate the catalyst. The powder obtained was subjected to an oven-drying process at 70 °C for 12 h, ensuring its viability for reuse.

The radical scavenging test is similar to that of photocatalysis, in which 20 mg of catalyst and 200 mL of diluted TC antibiotic solution were placed in a beaker. After 45 min in the dark, the following reagents were added to the solution: ethylenediaminetetraacetic acid (EDTA, 0.0144 g), isopropyl alcohol (IPA, 2.4 mL), and silver nitrate (AgNO_3_, 0.017 g). The procedure allowed the isolation and analysis of the distinct effects of each reactive species during the photocatalytic degradation process of the TC antibiotic.

## 3 Results and Discussions

### 3.1 X-ray Diffraction (XRD)

The XRD results for the NaTiNTs and TiNTs@CeW samples are presented in Figure 1a–b. The XRD pattern of NaTiNTs exhibits diffraction peaks at 2θ = 9.28°, corresponding to the interlayer distance, 2θ = 24.2°, attributed to the hydrogen-layered diagonal plane, and 2θ = 28° for sodium-layered diagonal planes.^23–25^ Additional peaks at 48° and 49° correspond to TiO_6_ octahedral planes, consistent with previous studies.^24,26–28^ These peaks can be indexed to the (200), (110), (211), (312), and (020) crystal planes, respectively, and confirmed in the ICSD file No. 015463.^23,24,28^ The low-intensity peaks in the NaTiNTs sample around 34° and 38° correspond to the (312̅) and (113) crystallographic planes, characteristic of titanate nanotubes.^23,26,29^

**Figure 1:**
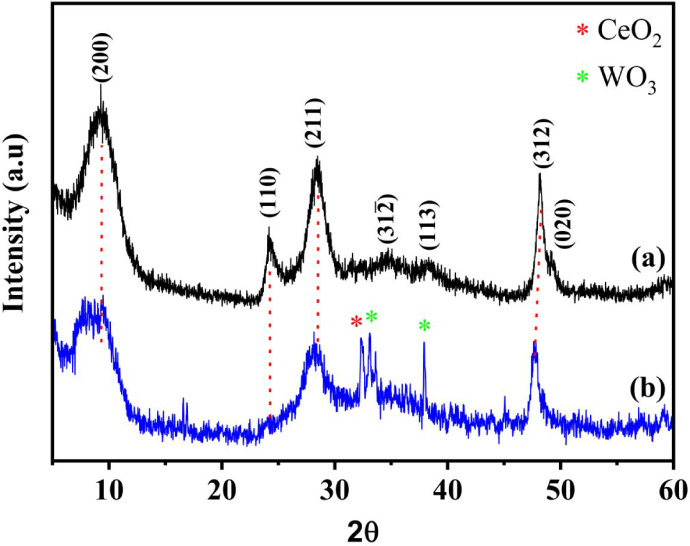
X-ray diffraction patterns of (a) NaTiNTs and (b) TiNTs@CeW samples. The main peaks are indexed according to the ICSD crystallographic pattern. The signals marked with (*) indicate the presence of the secondary phase attributed to CeO_2_ and WO_3_, respectively.

In Figure 1b, it can be observed that the peak corresponding to the interlayer spacing of the titanate nanotubes was shifted to 2θ = 8.69°, suggesting that strong surface interactions with CeW species play a role in promoting structural rearrangements within the titanate layers. This change was calculated using Bragg’s law.^30^

The interlayer distance for the titanate nanotubes is 0.77 nm for NaTiNTs and 0.82 nm for nanocomposite TiNTs@CeW. The absence of a peak around 2θ = 24.2° is observed in the TiNTs@CeW sample compared to the XRD pattern of the NaTiNTs sample. This is attributed to the presence of −OH groups on the surfaces of the TiNTs, which react with additional ions. These ions, when binding to the surface as oxides and/or hydroxides, can form a more amorphous layer over the nanotubes. This formation results in both a decrease and a shift of the diffraction peak around 24°, an effect that is also influenced by the larger ionic radius of the hydrated ions.^30–33^ The peak positions at 2θ = 32.2°, 33.09°, and 37.8° correspond, respectively, to the crystal planes (200) of the CeO_2_ phase and (111) and (201) of WO_3_, as indicated in the ICSD files No. 028709 and 32001.^20,34,35^ These results indicate the formation of the nanocomposite TiNTs@CeW after modification with Ce and W ions in the structure of the TiNTs.^32^

### 3.2 Morphology and Composition Analysis

The composition of the chemical elements was analyzed using EDS for the NaTiNTs and TiNTs@CeW samples, and the homogeneity of the materials was observed, where only the presence of the components that make up both samples was observed. Table 1 shows that the amount of Na remains practically constant, while the Ti content is lower in the nanocomposite TiNTs@CeW compared to NaTiNTs. This behavior may be related to the insertion of CeW nanoparticles on the surface of the sodium titanate nanotubes, whose amorphous layer reduces the Ti signal, as observed by the XRD results.^8,33^ The region mapped by EDS is shown in Figure SI01, available in the (Supporting Information SI).

**Table 1:** EDS Analysis Showing the Chemical Composition of the NaTiNTs and TiNTs@CeW Samples

Samples	Element	Weight (%)	Atomic (%)
NaTiNTs	Na	28.70	24.81
	Ti	15.35	6.38
	O	55.40	68.81
TiNTs@CeW	Na	27.70	23.12
	Ti	11.18	4.47
	O	60.40	72.33
	Ce	0.42	0.05
	W	0.14	0.01

The HRTEM analyses presented in Figure 2a–b for the NaTiNTs and TiNTs@CeW samples reveal that the tubular morphology of the nanotubes (TiNTs) was preserved from the nanocomposite TiNTs@CeW. In Figure 2a, the original NaTiNTs exhibit a typical tubular structure, characterized by external and internal diameters of approximately 9.7 and 3.9 nm, respectively, with multilayer containing 4 to 5 layers and an interlamellar distance of 0.78 nm consistent with the XRD result (Figure 1). The presence of a hollow cavity and characterization of nanotubes with *scroll*-type morphology are also observed, which confirm the predicted structural formation.^36^ In Figure 2b, the TiNTs@CeW maintains the tubular morphology, with external and internal diameters measured at 10.5 and 5.1 nm, respectively, and the interlamellar distance is 0.83 nm, corroborating the results of the XRD analyses.

**Figure 2:**
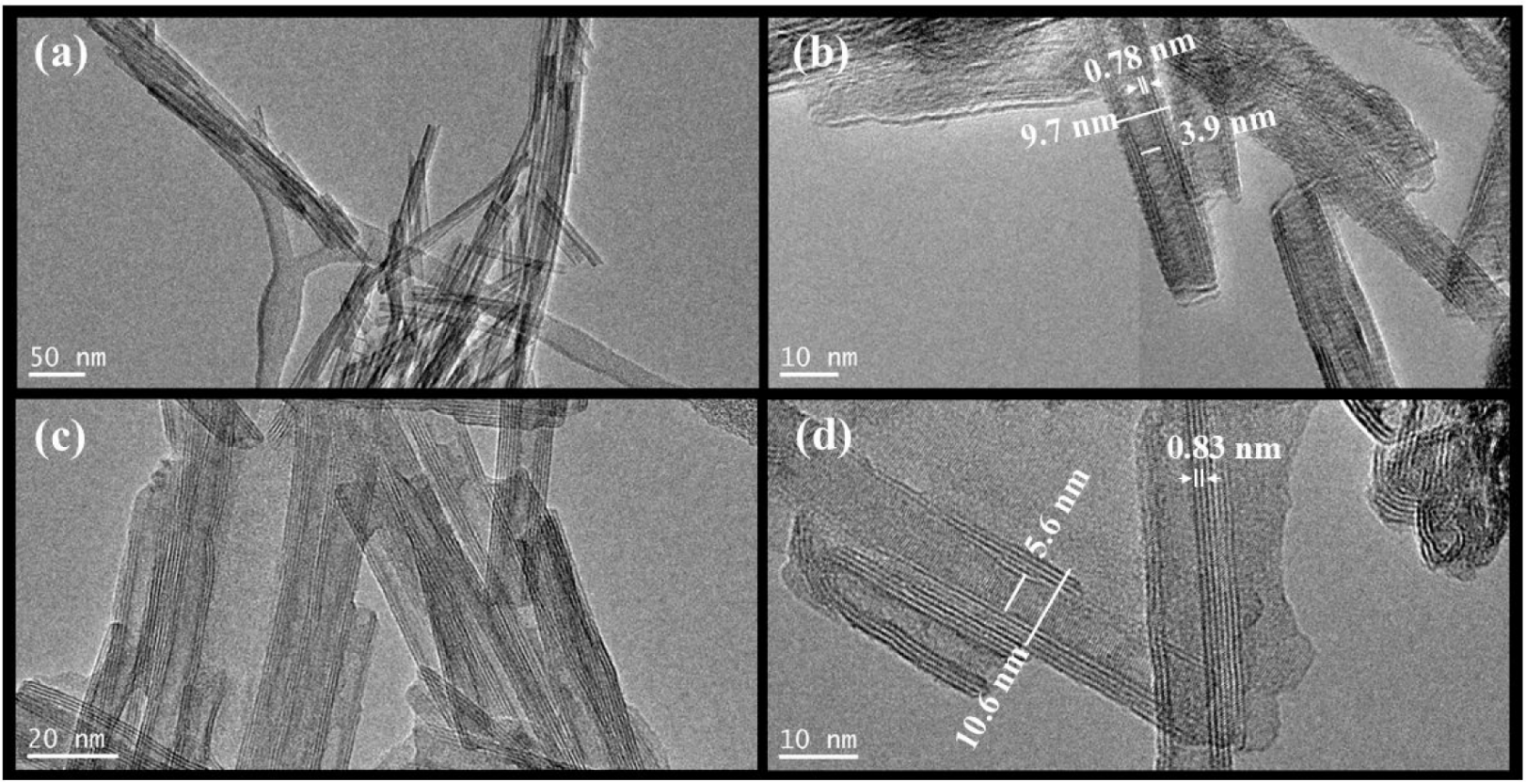
HRTEM images of (a) the NaTiNTs sample and (b) a magnification showing the interlayer distance and inner and outer thicknesses. (c) TiNTs@CeW sample (d) and magnification showing the interlayer distance and inner and outer thicknesses.

In addition to the TEM images, HAADF-STEM images were also acquired for the NaTiNTs and TiNTs@CeW samples, Figure 3a–d. In the TiNTs@CeW sample, bright spots were distributed along the nanotubes, which can be attributed to the deposition of species containing Ce and W on the surface of sodium titanate nanotubes. These spots present a high Z contrast about NaTiNTs, indicating the presence of elements with higher atomic numbers. According to Rodrigues et al.,^8^ the contrast in HAADF-STEM images is directly related to the atomic number of the elements present, being 11 for Na, 58 for Ce, and 74 for W. Additionally, it is possible to observe cluster regions in the TiNTs@CeW sample rich in cerium nanoparticles on the nanotube surfaces (Figure 3d), as some studies in the literature have demonstrated ion exchange and/or doping processes involving Ce^4+^ or Ce^3+^.^8,11,33,37^

**Figure 3:**
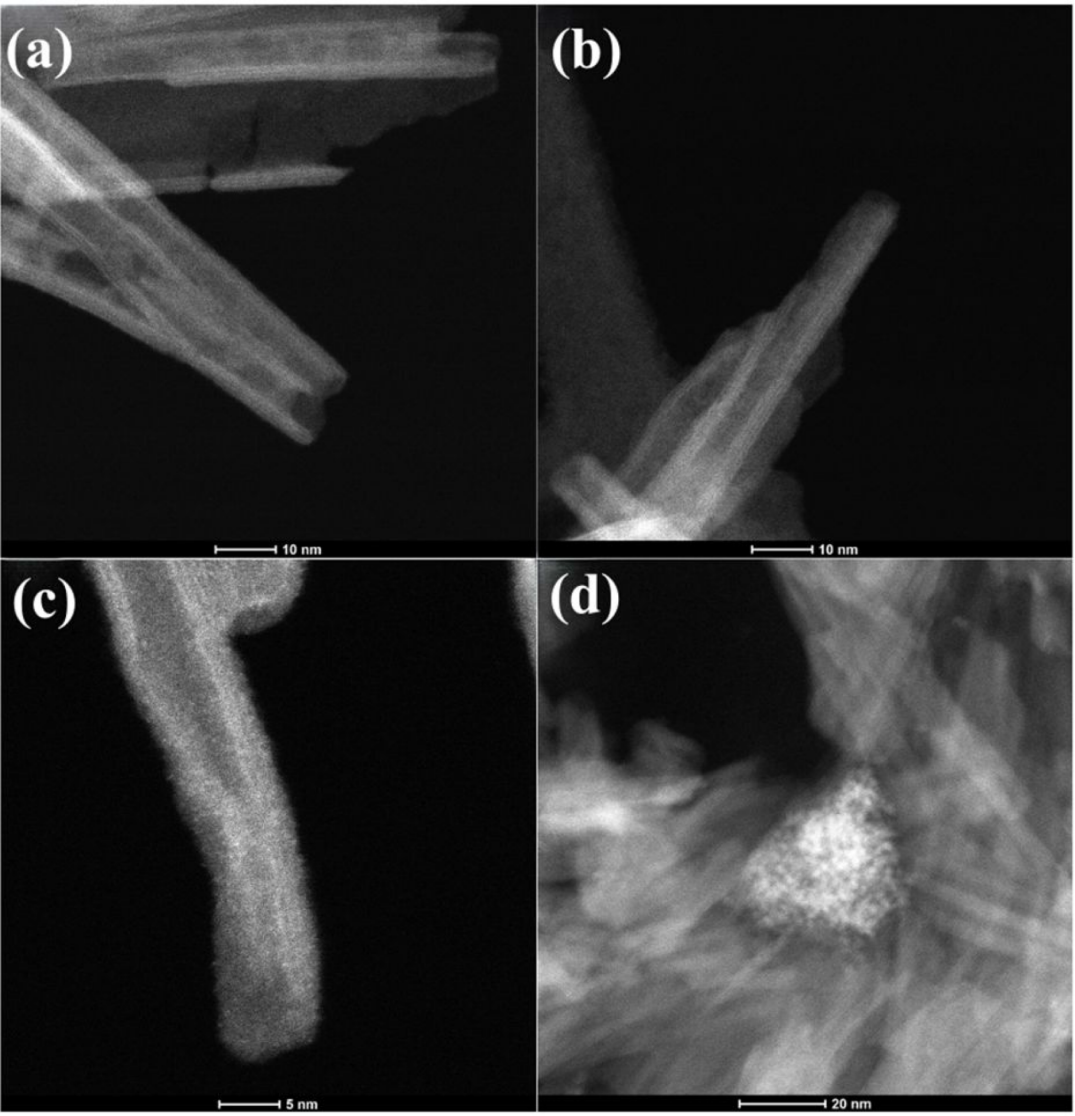
HAADF-STEM images of (a-b) NaTiNTs sample, (c) TiNTs@CeW sample, and (d) region with a high concentration of cerium (Ce), evidenced by the more intense contrast due to the higher atomic number of the element.

The chemical composition, stoichiometry, and oxidation states of the samples were examined by X-ray photoelectron spectroscopy (XPS). The results are shown in Table 2. The survey spectrum, recorded in the range of 0–1200 eV, confirmed the absence of contaminants and indicated that the samples were primarily composed of Na, Ti, O, Ce, and W, as shown in Figure 4a. XPS analysis was performed on two nanotube samples: NaTiNTs and TiNTs@CeW. The high-resolution spectra of W 4f, Ce 3d, O 1s, Ti 2p, and Na 1s, displayed in Figure 4b–f, reveal detailed chemical states that are not discernible in the survey scan.

**Figure 4:**
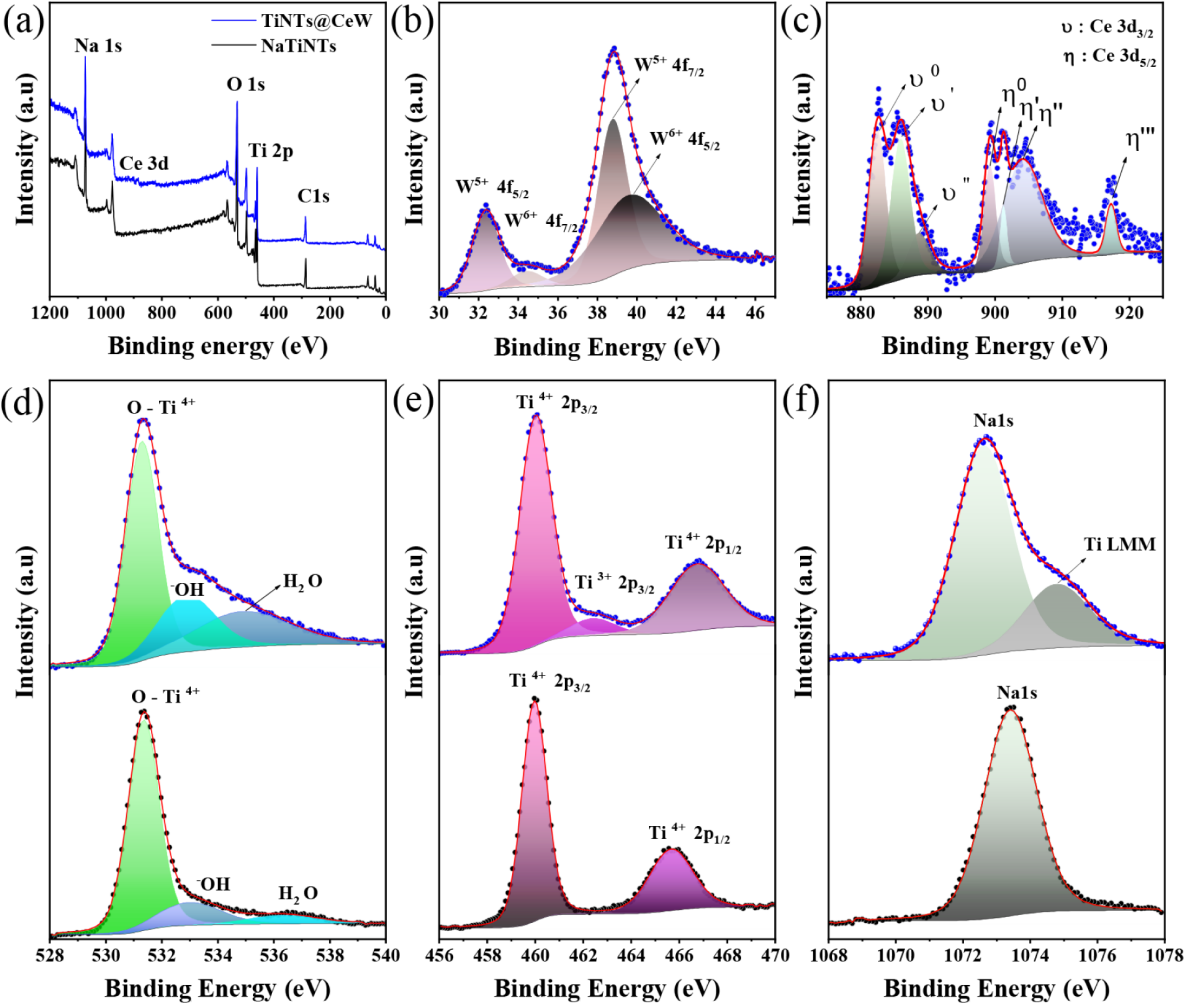
XPS spectra of NaTiNTs and TiNTs@CeW: (a) overall scan for NaTiNTs (black) and TiNTs@CeW (blue) samples; (b) W 4f region; (c) Ce 3d multiplets revealing Ce^3+^/Ce^4+^ mixed states; (d) O 1s peaks; (e) Ti 2p region; and (f) Na 1s signal.

**Table 2:** Surface Composition of Samples NaTiNTs, TiNT@CeW Determined by XPS Spectra Analysis

	Surface composition (%)				
Samples	Na 1s	Ti 2p	O 1s	W 4f	Ce 3d
NaTiNTs	11.45	14.01	43.71	–	–
TiNTs@CeW	16.45	2.22	43.34	0.12	0.21

The XPS results presented in Table 2 confirm the presence of Ti^3+^ and Ce^3+^ species after modification. These reduced species can act as charge-trap centers, influencing charge-carrier dynamics and linking defect states to charge separation efficiency. Simultaneously, they can contribute to increased photoluminescence intensity by acting as recombination centers and to increased Urbach energy, indicating greater structural disorder and the formation of localized states within the forbidden band. These data are consistent with EDS and XRD results, which suggest that modification of NaTiNTs with CeW introduced Ce/W and/or Na-containing species coating the nanotube surface.^38,39^ This effect reduces the intensity of the Ti signal detected by XPS, justifying the sharp decrease in Ti 2p, while Ce and W appear in relatively low concentrations. The increase in Na content suggests the presence of counterions or the surface removal/redistribution of species during the synthesis process.^40^ The difference between XPS and EDS directly highlights the surface modification of the nanotubes by CeW. The sharp drop in Ti in XPS (14.01% → 2.22%) indicates surface coverage, while the preservation of Ti in EDS (11.18%) confirms that the structural core remains intact. These results demonstrate that CeW species are predominantly concentrated on the nanotube surface.

Figure 4b shows the W 4f spectrum, which can be fitted to two groups of spin–orbit doublets of W 4f_7/2_ and W 4f_5/2_ with a separation distance of 2.1 eV, indicating two different oxidation states for the element W. The peaks at 39.7 and 34.42 eV can be assigned to the element W in the W^6+^ (45.35%) oxidation state, while the peaks at 32.3 and 38.8 eV can be assigned to the element W in the W^5+^ (54.68%) oxidation state. These binding energy results measured by W 4f_7/2_ and W 4f_5/2_ for the TiNTs@CeW sample indicate a tungsten oxide state.^41^

Figure 4c shows the Ce 3d spectrum. The peaks related to the 3d_5/2_ and 3d_3/2_ spin–orbit coupling are indicated as υ and η, respectively. The peaks labeled η″′ (917.1 eV), η″ (904.2 eV), η’ (901.2 eV), and υ’’ (888.8 eV) are characteristic of Ce^4+^ (47.26%), and the peaks labeled η^0^ (899.0 eV), υ’ (885.9 eV), and υ^0^ (882.5 eV) are characteristic of Ce^3+^ (64.08%) states.^30^ These results show that cerium is present in both oxidation states Ce^4+^ and Ce^3+^ in the sample TiNTs@CeW. The higher concentration of Ce^3+^ observed in the TiNTs@CeW sample may be a consequence of the interaction between CeW species and the nanotube surface, which can induce the formation of oxygen vacancies and favor partial reduction processes of Ce^4+^.^42^ Furthermore, the presence of W and the defective structure of NaTiNTs stabilize reduced states, which can result in a significantly higher Ce^3+^ content.^43^

Figure 4d shows the O 1s spectra, which were fitted by three Pseudo-Voigt singlets corresponding to distinct peaks were identified as (Ti^4+^-O) at around ∼531.0 eV, (^−^OH) at around ∼532.0 eV, and as (H_2_O) at around 536.0 eV for sample NaTiNTs, which correspond respectively to concentrations of 76.29%, 16.18%, and 6.88%.^44^ For sample TiNTs@CeW, an increase in the intensity of these bands is observed, related to the higher content of −OH (23.69%) groups and H_2_O (24.15%) after the modification of the NaTiNTs, followed by a reduction in the concentration of Ti^4+^-O (52.28%). This behavior can be attributed to the interaction of CeW with the nanotube surface, leading to the possible adsorption of hydrated species and the formation of hydroxyl groups.

Figure 4e shows the Ti 2p spectra. A Shirley background was also extracted. In NaTiNTs, the peaks were fitted by applying spin–orbit doublets, one for Ti^4+^ 2p_3/2_ (67.32%) and the other for Ti^4+^ 2p_1/2_ (32.68%).^45^ Meanwhile, for a sample of TiNTs@CeW, the peaks were fitted by three Pseudo-Voigt singlets corresponding to distinct peaks were identified as Ti^4+^ 2p_3/2_ (62.44%) at around ∼459.4 eV, Ti^3+^ 2p_3/2_ (6.49%) at around ∼461.5 eV, Ti^4+^ 2p_1/2_ (31.07%) at around ∼465.2 eV.^45,46^ The binding energy (BE) difference between the peaks of the Ti 2p_1/2_ and Ti 2p_3/2_ spin–orbit doublet was estimated at approximately 5.8 eV, confirming the electronic state of Ti. This value is characteristic of the Ti^4+^-O bonds present in pure TiO_2_, as also analyzed by the XPS spectrum of the O 1s region.^45,46^

Figure 4f shows the Na 1s spectra, which were fitted using two Pseudo-Voigt singlets with a Shirley background. In the sample of NaTiNTs, the peak at approximately 1073 eV corresponds to Na 1s.^30^ In the TiNTs@CeW sample, the peak is at approximately 1072 eV, associated with Na 1s, while the peak around 1074 eV corresponds to the Ti Auger band.^47^

### 3.3 Dynamic Light Scattering (DLS)

In Table 3, the ζ-potential measurements and the average size distributions of the nanoparticles are characterized using dynamic light scattering (DLS) measurements and the polydispersity index (PDI), which is a unitless value that measures the width of the particle size distribution. Zeta potential analysis showed that the aqueous suspension of titanate nanotubes was negatively charged (−32.9) for NaTiNTs and (−32.7) for TiNTs@CeW. This can be explained by the electrostatic interaction between the nanotubes and the CeW nanoparticles in the aqueous solution, possibly related to the small size and uniform distribution of these nanoparticles on the surface of the nanotubes, as evidenced by the difference in the hydrodynamic diameter and polydispersity index values (Table 3).^42^ The hydrodynamic diameter values presented in Table 3, obtained by DLS, assume spherical particles for the size calculation. For the samples in question, which have a nonspherical morphology, these values should be interpreted as apparent size, mainly reflecting the longitudinal dimension of the particles. The change in hydrodynamic diameter values indicates that the modification reduces aggregation and alters the dispersion state of the nanotubes, probably because of modifications in the surface charge and/or the solvation layer. This improvement in colloidal stability and more uniform size distribution may contribute to better catalytic performance and enhanced electronic properties.^48,49^

**Table 3:** Zeta Potential Values, Hydrodynamic Diameter, and Polydispersity Index

Samples	Zeta potential (mV)	Hydrodynamic diameter (nm)	PDI
NaTiNTs	–32.9	213.1	0.823
TiNTs@CeW	–32.7	243.3	0.332

### 3.4 FTIR and Raman Spectroscopy

The FTIR spectra in Figure 5a–c correspond to the NaTiNTs and TiNTs@CeW samples. A broadband at ∼3400 cm^–1^ and a narrow band at ∼1600 cm^–1^ are attributed to water and hydroxyl groups, respectively. The band at ∼437 cm^–1^ for the NaTiNTs sample and ∼443 cm^–1^ for the TiNTs@CeW sample is attributed to the symmetric Ti–O–Ti vibration of the TiO_6_ octahedron. The bands around 892 cm^–1^ (NaTiNTs) and 887 cm^–1^ (TiNTs@CeW) are related to the symmetric Ti–O stretching.^50,51^ Regarding the NaTiNTs sample, a shift of the bands to lower wave numbers is observed, which is associated with the presence of CeW in the TiNTs@CeW sample. For the TiNTs@CeW sample, the band around 849 cm^–1^ is due to the W–O–W stretching vibration modes characteristic of tetrahedral tungstate (WO_4_^2–^).^52–54^ Naderi et al.^17^ report that the characteristic bands of the scheelite-type structure are between 950 and 460 cm^–1^. According to Erdem et al.,^53^ the shift of the peaks within this spectral range may indicate the formation of the W–O–Ce bond, which would explain the shifts observed in the bands presented in this work. It can be observed that the incorporation of CeW into the nanotube surface resulted in a noticeable decrease in the intensity of the vibrational modes located at approximately 892 cm ^–1^ and 474 cm^–1^, originally observed in NaTiNTs. The narrow peak observed around 1443 cm^–1^ indicates the presence of water molecules adsorbed on cerium nanoparticles.^55^

**Figure 5:**
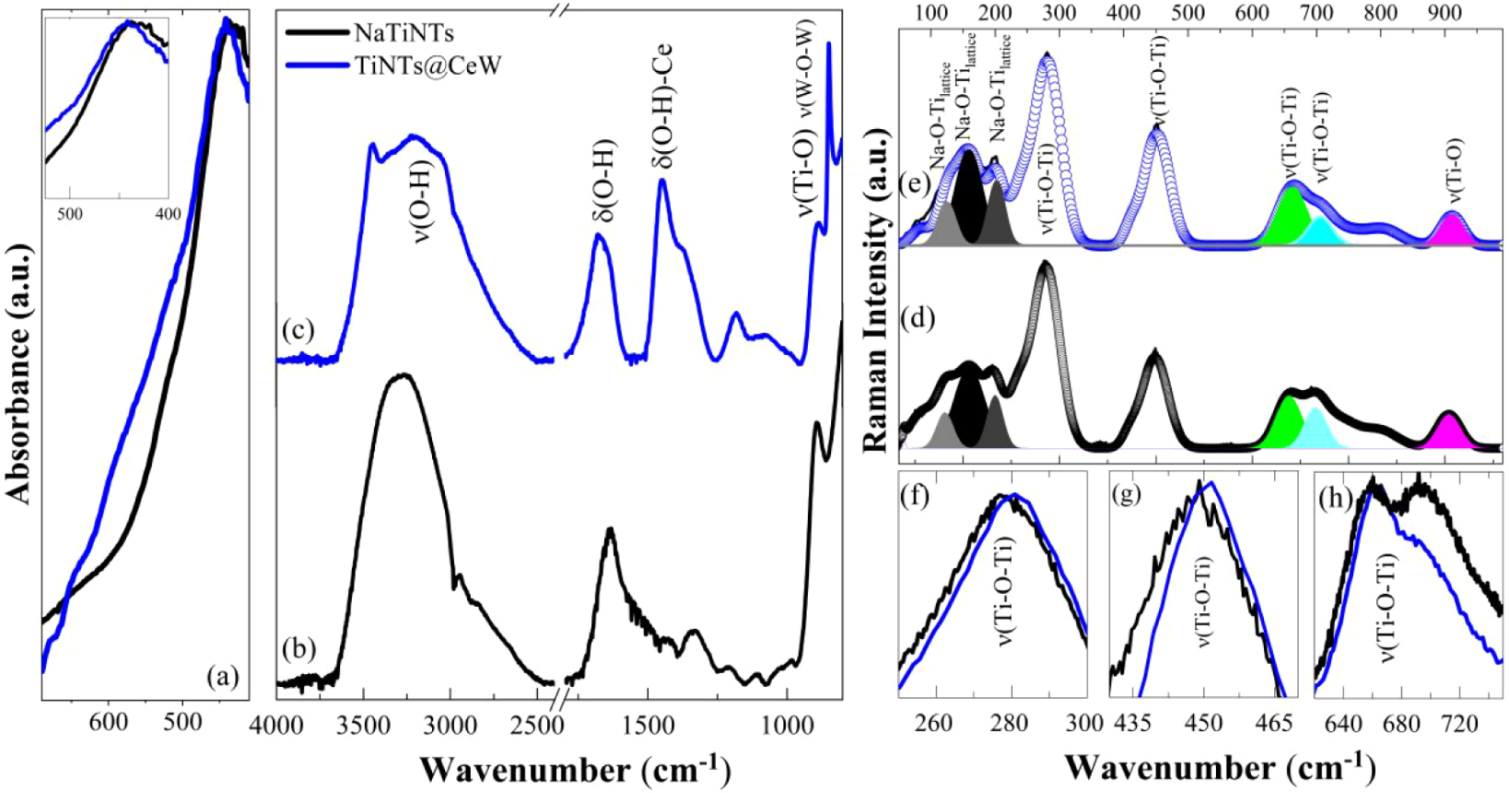
FTIR and Raman spectra of samples NaTiNTs and TiNTs@CeW with your assignments. (a) FTIR spectrum of the sample NaTiNTs of the regions (a) 700–400 cm^–1^ and (b-c) 4000–800 cm^–1^. (e-d) Raman spectrum of samples NaTiNTs and TiNTs@CeW. Graphs (f-h) show magnifications of specific Raman regions, highlighting the shift in ν­(Ti–O–Ti) modes. Both exhibit bands attributed to the characteristic vibrations of sodium titanate nanotubes. (ν is the stretching vibration, and δ is the angular deformation vibration).

The Raman spectra of NaTiNTs and TiNTs@CeW are shown in Figure 5-d–e. In Figure 5d, we observe that the Raman spectrum of the NaTiNTs exhibits characteristic vibrational modes of sodium titanium nanotubes, with modes located at approximately 119, 154, and 197 cm^–1^ (gray and black colors) that can be attributed to lattice modes and stretching vibrations corresponding to the Na–O–Ti bonds. Meanwhile, the modes identified in 277, 449, 659 (green color), and 695 cm^–1^ are associated with the vibrations of Ti–O–Ti, which are present in the (TiO_6_) octahedral of the tubular walls.^51,56^ The mode around 906 cm^–1^ (magenta color) is attributed to the stretching of nonbridging terminal Ti–O bonds.^11,30,33^ The vibrational modes identified in this work are consistent with studies conducted in the literature.^8,25^

The intensity corresponding to the mode around 195 cm^–1^ in the NaTiNTs (Figure 5d) was lower than that observed for the TiNTs@CeW (Figure 5e). The shift of the peaks around 118, 158, and 200 cm^–1^ associated with lattice modes (Na–O–Ti) corroborates the modification of NaTiNTs with CeW.^32,57^ The modes around 277 and 448 cm^–1^ in the NaTiNTs cm^–1^ shifted to 281 and 451 cm^–1^ due to modifications of different cations (Figure 5f–g), which slightly modify the intensity and position of the Raman modes associated with Ti–O–H/Na vibrations.^32^ In Figure 5g, the reduction in Raman peak intensity around 690 cm^–1^ (cyan color) can be attributed to the adsorption of hydrated species and the formation of hydroxyl groups on the surface, induced by the modification of sodium titanate nanotubes with CeW. These processes can promote partial substitution of bridging oxygens by terminal oxygens (Ti–OH), increasing the local structural disorder in the TiO_6_ octahedra.^58^ As a consequence, damping of the collective Ti–O–Ti vibrational modes occurs, and therefore, the Raman peak intensity decreases by ∼690 cm^–1^. These results are in agreement with XPS analyses, which indicated a reduction in the Ti concentration on the surface of the nanotubes. The peak shift around 913 cm^–1^ for higher wavenumbers, and the increase in its full width at half height (fwhm) jointly indicate structural modifications that occurred in the titanate structures, consistent with the findings reported by Méndez-Galván et al.^32^ The FWHM values were obtained from the deconvolution analysis of the Raman spectra, showing 39.26 cm^–1^ for the NaTiNTs sample and 34.52 cm^–1^ for the TiNTs@CeW sample.^8,32,57^ These results closely match the literature and exhibit strong consistency with the XRD patterns.^23,32,57,59^

### 3.5 Ultraviolet–Visible Spectroscopy (UV–Vis)

Figure 6a–b shows the UV–vis spectra of the NaTiNTs and TiNTs@CeW samples. It is observed that the NaTiNTs sample, Figure 6a, exhibits an absorption band in the ultraviolet region (below 380 nm), as noted by Viana et al.^24^ report on what limits the use of titanate nanotubes as heterogeneous catalysts that operate with excitation in the visible region. The UV–vis spectrum of the TiNTs@CeW sample (Figure 6b) shows a shift of the absorption band to around 450 nm, indicating excitation into the blue-violet region. The red shift of the absorption band edge may be caused by the modification of nanoparticles of CeW, which alters the energy of the material’s band gap.^24,33^

**Figure 6:**
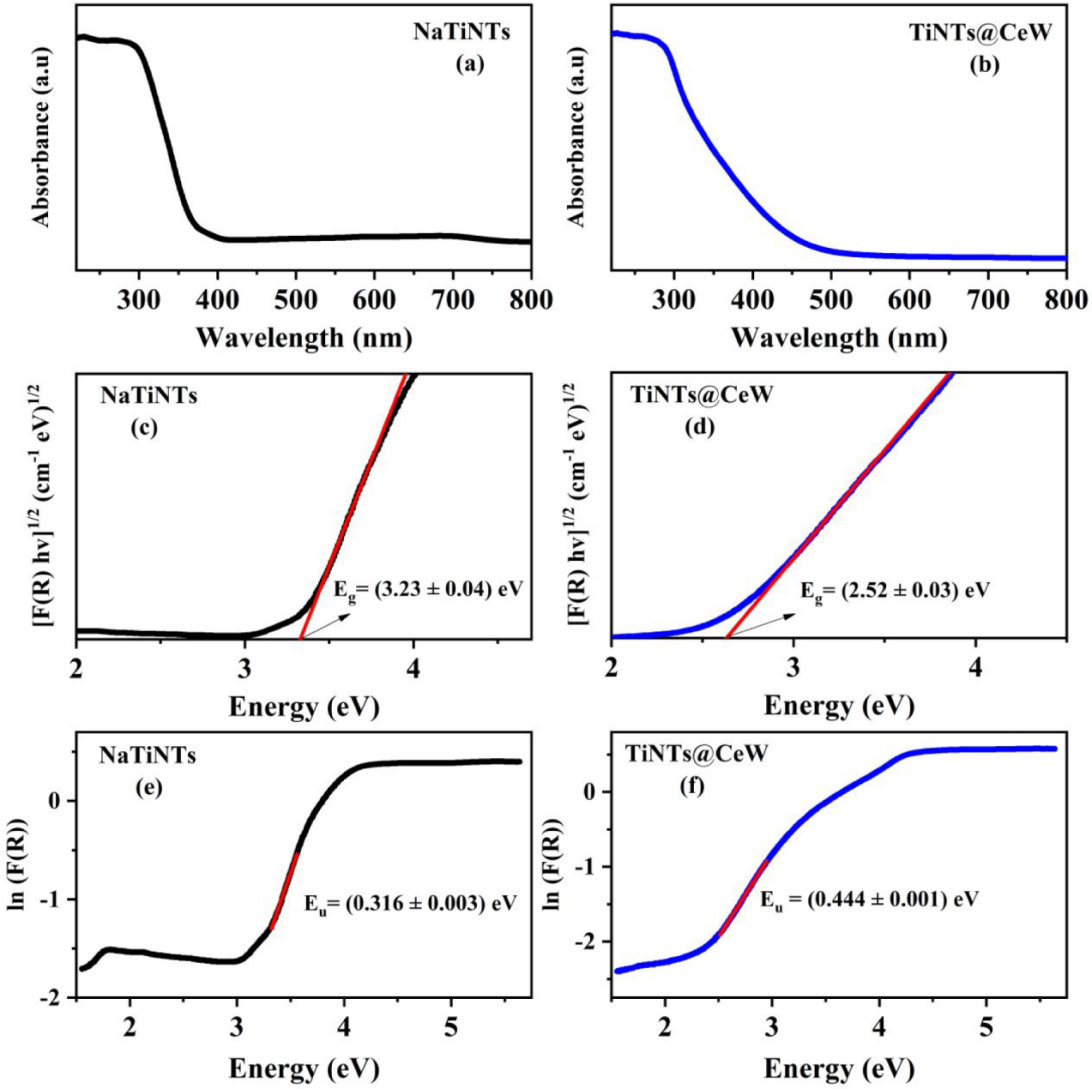
(a-b) UV–vis spectra of NaTiNTs and TiNTs@CeW. (c-d) Graph of [F­(R) hυ]^1/2^ versus Energy (eV) for NaTiNTs and TiNTs@CeW samples. (e-f) Urbach energy for NaTiNTs and TiNTs@CeW samples.

Figure 6c–d shows a decrease in the band gap energy of the TiNTs@CeW sample, which may primarily be attributed to modification with nanoparticles of CeW. The band gap energy was calculated using the method proposed by Kubelka–Munk^60^ from the diffuse reflectance spectrum of the samples, converting the vertical axis (y) to F­(*R*_∝_) and thus expressed in the following form;

1 $$F\left(R_{\propto }\right)=\frac{{\left(1-R_{\propto }\right)}^{2}}{2R_{\propto }}=\frac{k}{s}$$

where *R*_∝_ is the diffuse reflectance of the sample, *k* is an apparent absorption coefficient, and *s* is the apparent scattering coefficient. To measure the optical gap energy *E_g_* in structures, the diffuse reflectance of the sample is plotted as a function of photon energy (*hv*) in electron volts (eV), known as a Tauc plot,^61^ as indicated in eq 2, which uses terms k = 2∝ e *s* = C_2_.

2 $${[F\left(R_{\propto })hv\right)]}^{n}=C_{2}\left(hv-E_{g}\right)$$

where *α* is the absorbance, *hv* is energy, *E_g_* is the band gap, and *n* is the experimental coefficient. The band gap was estimated from the TiNTs@CeW sample at (2.52 ± 0.03) eV, which is lower than the band gap of (3.23 ± 0.04) eV for the NaTiNTs sample.

Optical absorption spectroscopy can be used to analyze defects in the system, which can be estimated in terms of the Urbach energy parameter *E*_u_.^28^ Modification of TiNTs with metallic ions may have introduced localized defect states into the original structure of the titanate.^28,61^ These defect states lead to a reduction of the bands in the forbidden energy band regions, consequently resulting in a decrease in the forbidden band widths. These band tails are called Urbach tails and are characterized in terms of Urbach energy, indicating an estimate of the overall defects in the system.^27,28,62,63^ The Urbach energy can be calculated according to the following expression:^60^

3 $$\alpha \left(\omega \right)=a_{0}exp\left(\frac{\hbar v}{E_{u}}\right)$$

where α_0_ is one constant, E_u_ is the Urbach energy, and the other variables have been enumerated in this work.

Figure 6e−) shows the Urbach energy graphs for the NaTiNTs and TiNTs@CeW samples. It can be observed that the Urbach energy increased for the TiNTs@CeW sample compared with the NaTiNTs. This may be related to the increase in oxygen vacancies in the NaTiNTs caused by the insertion of CeW, complementing the information obtained from the band gap and increasing the red shift of possible band-tail and tail–tail transitions.^27^ These results are in agreement with studies on the modification of NaTiNTs using various materials, such as Ce,^24^ Cr,^63^ Mg,^28^ and Co,^27^ demonstrating that the synthesis process employed was efficient, as indicated by the analysis of the data obtained from the applied techniques.

### 3.6 Photoluminescence Properties

Figure 7a–c shows the photoluminescence (PL) spectra of the NaTiNTs and TiNT@CeW samples. Photoluminescence spectroscopy allows the investigation of the physical characteristics of semiconductors, such as defects, surface states, and the separation and recombination of photogenerated charges.^64^

**Figure 7:**
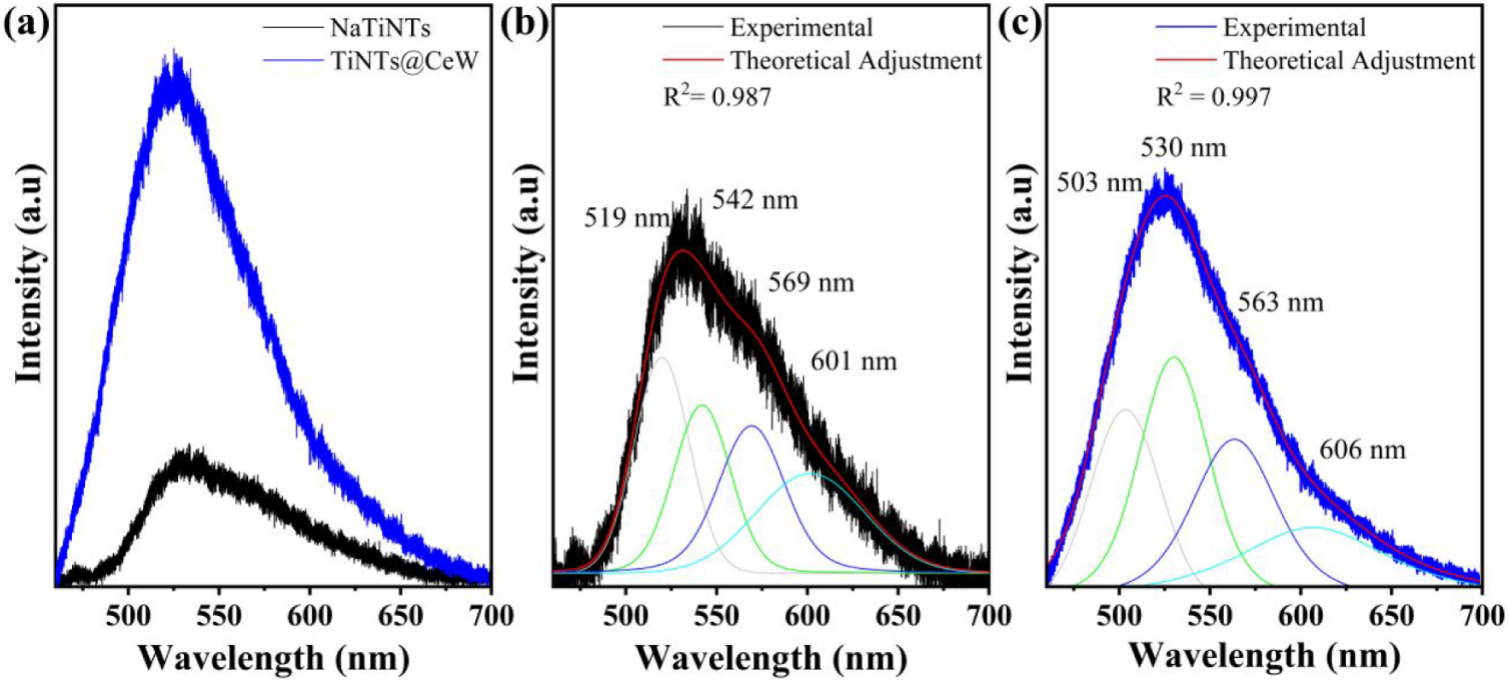
Photoluminescence (PL) emission spectra of NaTiNTs and TiNTs@CeW samples (a) and deconvolved spectra using Voigt function fitting for NaTiNTs (b) and TiNTs@CeW samples (c).

In Figure 7a, an increase in PL intensity is observed for TiNTs@CeW compared to NaTiNTs. According to Mrabet et al.,^65^ the increase in photoluminescence intensity in systems rich in structural defects can be attributed to a higher concentration of oxygen vacancies and localized electronic states, which act as radiative recombination centers, intensifying photoluminescence. Simultaneously, these defects function as temporary nonradiative charge traps that retard electron–hole recombination in the bulk, directing charges toward the surface. In the present study, this dual effect is corroborated by the increase in the Urbach energy, indicative of greater electronic disorder induced by CeW modification. These sub-band gap states not only intensify emission but also promote surface reactions, increasing the generation of reactive oxygen species and, consequently, photocatalytic activity.

According to Oliveira et al.,^64^ the broad PL band observed in both samples is characteristic of multiphonic processes, in which emission occurs through multiple pathways due to the high density of electronic states caused by defects within the band gap region of the NaTiNTs. Figure 7b–c shows the Voigt function fit of the photoluminescence spectra for the NaTiNTs and TiNTs@CeW, with four emission peaks. The NaTiNTs exhibit peaks at ∼519, 542, 569, and 601 nm, while the TiNTs@CeW show bands at ∼503, 530, 563, and 606 nm. The modification with CeW induces a shift in emissions, mainly toward shorter wavelengths, associated with the redistribution of defect states in the band gap, related to oxygen vacancies, Ce^3+^/Ce^4+^ states, and electron–phonon coupling.^64,66^

### 3.7 Tetracycline Photodegradation Test

The tetracycline photodegradation experiments (40 ppm) using NaTiNTs and TiNTs@CeW at a photocatalyst concentration of 0.1 g L^–1^ demonstrated significant differences in efficiency between the tested samples and are shown in Figure 8a–b. The high initial concentration of the pollutant represents a substantial challenge for photocatalytic degradation, making the material’s efficiency a key factor in determining its feasibility for real-world applications.^67^

**Figure 8:**
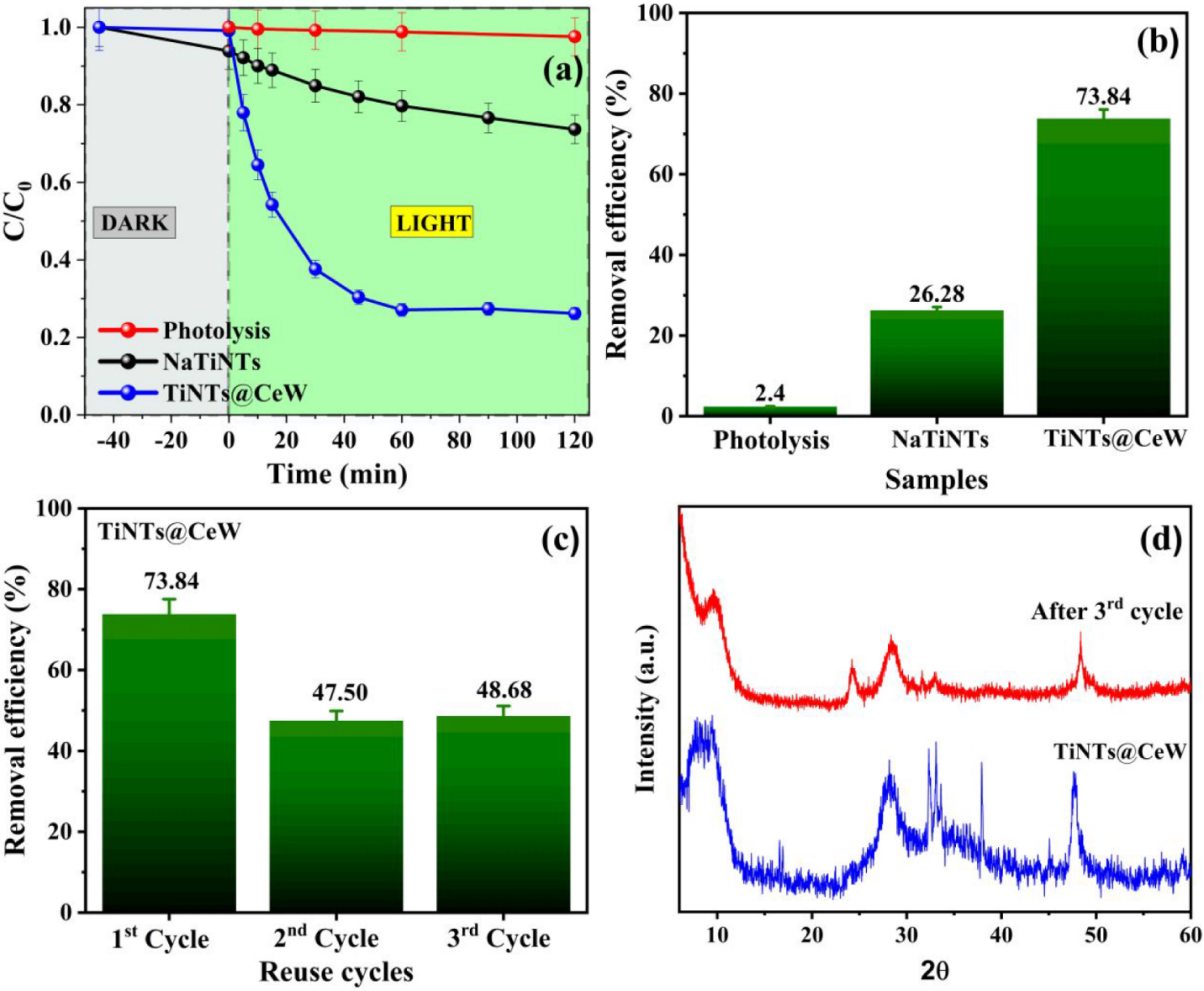
(a) Concentration versus irradiation time, (b) removal efficiency of photolysis samples, (c) TiNTs@CeW (0.1 g L^–1^) reuse study using TC (40 ppm), and (d) XRD patterns before and after the third reuse cycle.

The NaTiNTs sample exhibited a removal efficiency of only 26.28% after 120 min of irradiation, indicating limited photocatalytic activity. This lower performance is corroborated by the low reaction rate constant (*k* = 0.0020 min^–1^, R^2^ = 0.95407), which resulted in a high half-life (345.17 min). This behavior may be related to the low efficiency in generating reactive oxygen species (ROS).

In contrast, the TiNTs@CeW sample exhibited significantly superior performance, achieving a degradation efficiency of 73.84% within the same period. The reaction rate constant (k = 0.0105 min^–1^, R^2^ = 0.92952) was approximately five times higher than that of NaTiNTs, reflecting a more favorable kinetics.^22^ The reduced half-life (66.05 min) indicates a significantly faster degradation rate. These results suggest that the structural and chemical modification of TiNTs@CeW led to an improvement in photocatalytic properties, possibly due to a greater UV–vis radiation absorption capacity resulting from its lower band gap value (2.52 ± 0.03 eV) and a better electron–hole pair separation due to the insertion of CeW into the NaTiNTs structure.^30^

TiNTs@CeW reuse tests were conducted to evaluate their structural stability and photocatalytic performance over multiple cycles, as shown in Figure 8c. In the second cycle, the tetracycline degradation efficiency dropped to 47.50%, while in the third cycle, there was a slight recovery to 48.68%. This initial reduction, followed by efficiency stabilization, may be related to the accumulation of degradation byproducts on the catalyst surface, partially blocking the active sites and hindering interactions with the reactants.^22,68^ The comparative analysis of the XRD diffractograms (Figure 8d) corroborates this hypothesis, showing that although the crystalline structure of the TiNTs@CeW was preserved after three reuse cycles, there was the appearance of the peak at 24.2°, related to the diagonal plane (hydrogen-lamella) present in the NaTiNTs sample.^23,69^ TEM images of the sample after reuse are shown in Figure SI02 (Supporting Information), confirming the maintenance of the tubular morphology, corroborating that the initial drop in performance is related to surface effects and not to structural changes in the material. Following the reuse cycles (upper curve), the appearance of this peak suggests the removal of the amorphous layer in the nanotubes present in the sample, with the insertion of CeW, possibly also composed of organic residues and ions from the reaction medium.^30^ This behavior may be associated not only with the removal of the amorphous layer during reuse but also with the adsorption/desorption of reaction intermediates or byproducts accumulated on the surface during the photocatalytic process. Such reversible interactions on the surface explain the observed cyclic stability and the changes in the intensity of the X-ray diffraction peak, highlighting the dynamic nature of the catalyst surface under operating conditions.^22^

Despite this reduction, maintaining a degradation efficiency above 45% throughout the cycles demonstrates that TiNTs@CeW exhibits good stability, making it a promising candidate for sustainable photocatalytic applications. Thus, the performance difference between the samples highlights the importance of structural and chemical modifications with CeW in enhancing the photocatalytic efficiency, particularly under high pollutant concentrations and low photocatalyst loading.

The data presented in Table 4 highlight the relevance of TiNTs@CeW in achieving a balanced performance between degradation efficiency and operational sustainability compared to previously reported photocatalysts for tetracycline removal. While systems such as Ag-TNT (83.1%) rely on noble metals and higher-power light sources (250 W), and OTNs require a catalyst dosage six times greater (0.625 g L^–1^) to achieve lower removal efficiency (60.6%), TiNTs@CeW attained 73.84% degradation with a competitive kinetic constant (1.05 × 10^–2^ min^–1^) using only 0.10 g L^–1^ under a low-power mercury lamp (120 W). Moreover, compared to 1CeTiO_2_–Sep, TiNTs@CeW exhibited improved reaction kinetics under an equivalent pollutant concentration (40 ppm). These results indicate that the modification of titanate nanotubes with cerium tungstate effectively enhances photocatalytic efficiency while maintaining reduced catalyst loading and moderate energy input, supporting its potential applicability in antibiotic-contaminated wastewater treatment.

**Table 4:** Comparison of Parameters and Photocatalytic Performance of Different Materials in the Degradation of Tetracycline

Sample	Concentration (gL^‑1^)	Pollutant concentration (ppm)	Kinetic constant k(min^‑1^)	Light source	Removal efficiency (%)	Ref
TiO_2_-450	0.15	Tetracycline (40 ppm)	1.22 × 10^–2^	Xenon lamp (300 W)	77.3	^70^
NaTiNTs-2NH	0.10	Tetracycline (40 ppm)	8.5 × 10^–3^	Hg Lamp (120 W)	65.80	^51^
OTNs	0.625	Tetracycline (15 ppm)	1.23 × 10^–2^	Xenon lamp (500 W)	60.6	^71^
Ag-TNT	0.05	Tetracycline (50 ppm)	7.59 × 10^–2^	Philips 250 W	83.1	^72^
1CeTiO_2_–Sep	0.5	Tetracycline (40 ppm)	4.21 × 10^–3^	Lamp-Visible (120 W)	70.45	^22^
TiNTs@CeW	0.10	Tetracycline (40 ppm)	1.05 × 10^–2^	Hg Lamp (120 W)	73.84	This work

To elucidate the photocatalytic mechanism, radical scavenger experiments were performed (Figure 9). Under conditions without scavengers, the sample showed 73.84% tetracycline degradation, reflecting the combined action of holes (h^+^), electrons (e^–^), and hydroxyl radicals (·OH), generated after excitation of the photocatalyst by UV radiation (eq 4). For the study of scavengers, after reaching adsorption equilibrium, ethylenediamine tetraacetic acid (EDTA) (0.0144 g), isopropyl alcohol (IPA) (2.4 mL), and silver nitrate (AgNO_3_) (0.017 g) were added to the solution, as described in previous studies.^22,73^

**Figure 9:**
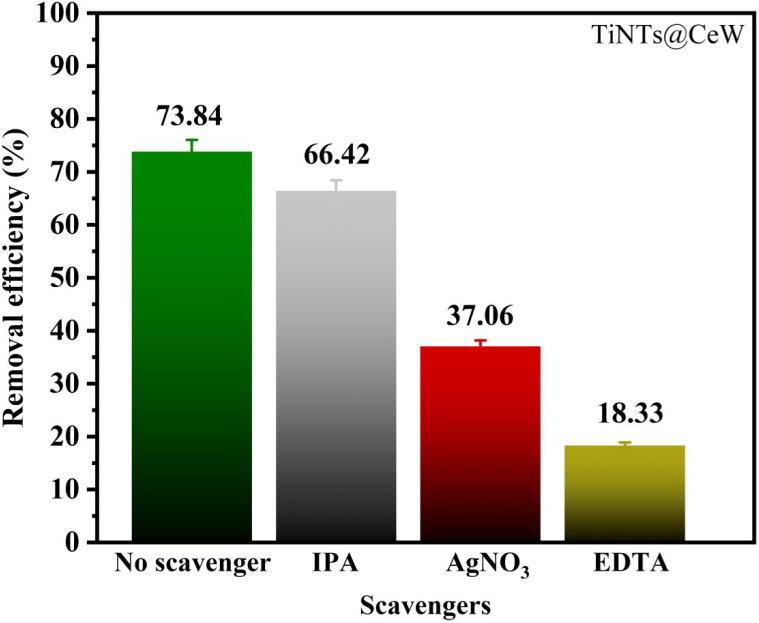
Scavenger assays with three types related to isopropyl alcohol (IPA), silver nitrate (AgNO_3_), and ethylenediaminetetraacetic acid (EDTA) during the photodegradation of the antibiotic TC using cerium tungstate-modified sodium titanate nanotubes (TiNTs@CeW) as a catalyst.

The addition of EDTA, which acts as a hole scavenger, drastically reduced the efficiency to 18.33%. This sharp drop indicates that holes play a dominant role in the oxidative process, participating both in the direct oxidation of adsorbed tetracycline (eq 5) and in the generation of hydroxyl radicals by water oxidation (eq 6).^22^ Thus, the inhibition of h^+^ suppresses both the direct oxidative pathway and the indirect radical-mediated pathway, confirming that hole-driven oxidation is the primary degradation route. Furthermore, these results corroborate the photoluminescence analysis, in which the increase in PL intensity of the TiNTs@CeW sample was associated with a higher concentration of oxygen vacancies and localized electronic states. These defects act as radiative emission centers and temporary charge traps, favoring charge separation and consequently contributing to the improvement of photocatalytic activity.

In the case of isopropyl alcohol (IPA), a specific •OH scavenger (eq 7), the efficiency decreased to 66.42%, revealing that hydroxyl radicals act as a complementary, but not predominant, oxidative pathway. The moderate inhibition observed confirms that •OH mainly participates in secondary oxidation steps following the initial hole attack on tetracycline. The presence of AgNO_3_, an electron scavenger by selective e^–^ capture (eq 8), reduced degradation to 37.06%. This response demonstrates that electrons also make a relevant contribution, especially because their removal favors recombination with h^+^, decreasing the availability of vacancies and the consequent formation of hydroxyl radicals. Therefore, electrons contribute to maintaining the surface redox balance, indirectly sustaining the oxidative degradation cycle.

Thus, the synergistic contribution of these reactive species, as described by eqs 4 [Disp-formula eq8], underscores the robustness of the TiNTs@CeW photocatalytic pathway. This mechanism supports the applicability of the material to advanced oxidation processes for environmental remediation.

4 $$TiNTs@CeW+hv\to {e^{-}}_{\left(CB\right)}+{h^{+}}_{\left(VB\right)}$$

5 $${h^{+}}_{\left(VB\right)}+{Tetracycline}_{\left(ads\right)}\to {TC}^{•+}\to degradation\;intermediates$$

6 $${h^{+}}_{\left(VB\right)}+H_{2}O/OH^{-}\to •OH$$

7 $$•OH+{Tetracycline}_{\left(ads\right)}\to oxidized\;intermediates$$

8 $${e^{‐}}_{\left(CB\right)}+O_{2}\to {O_{2}}^{•‐}$$

Superoxide radicals participate in secondary oxidation reactions, assisting in the progressive breakdown of tetracycline. The integrated photocatalytic mechanism is schematically illustrated in Figure 10.

**Figure 10:**
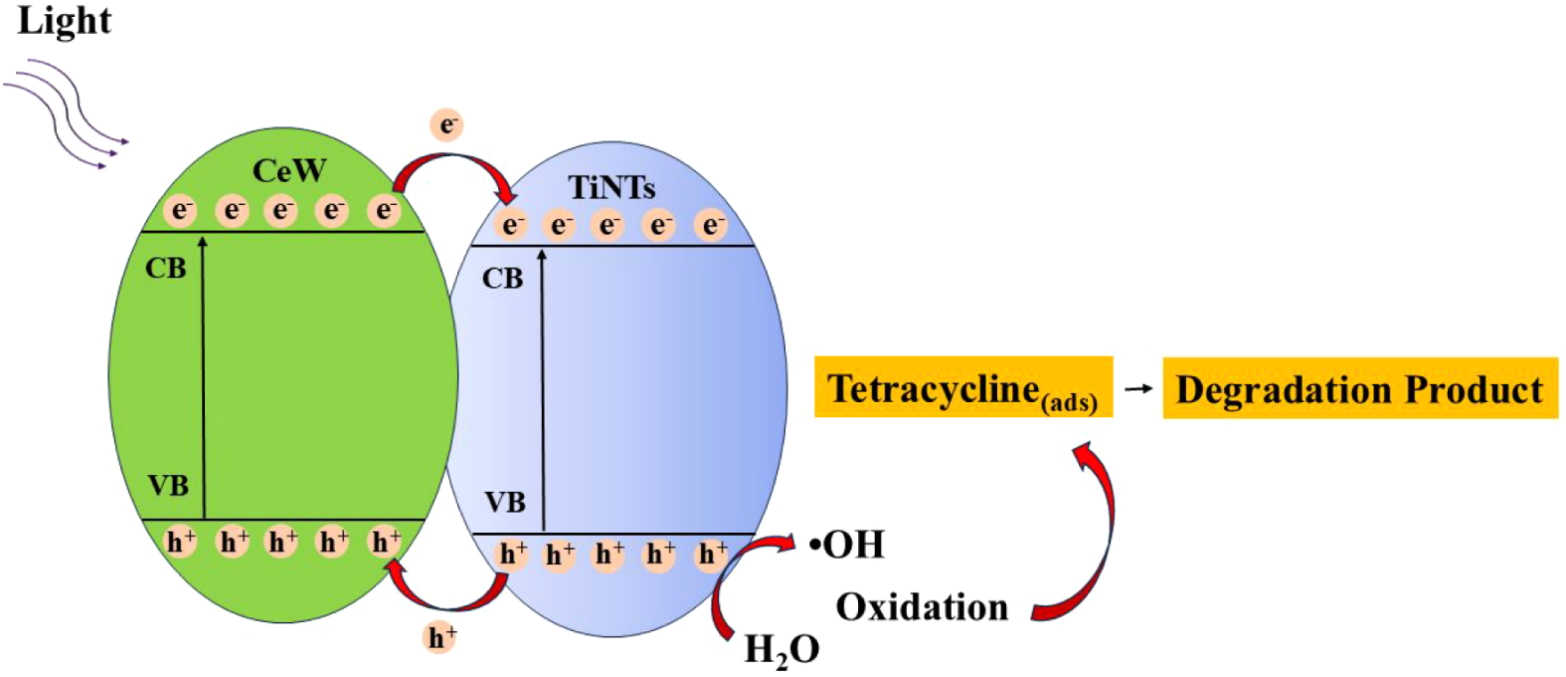
Schematic illustration of the proposed photocatalytic mechanism for tetracycline degradation over TiNTs@CeW under light irradiation.

In addition to the high photocatalytic degradation efficiency demonstrated in this study, further investigations into mineralization pathways and environmental safety assessments will broaden the practical applicability of this material in water-treatment systems. The identification of degradation intermediates using advanced analytical techniques, such as liquid chromatography coupled with mass spectrometry (LC-MS), will allow for a deeper understanding of the reaction mechanism and the degree of mineralization. Furthermore, comprehensive ecotoxicity assessments of the treated solution, including seed germination assays and microbial activity tests, are essential to validate its environmental compatibility. These complementary analyses represent a promising next step toward implementing this photocatalyst in sustainable wastewater-remediation technologies.

## 4 Conclusions

The structural and spectroscopic analyses confirmed the successful modification of sodium titanate nanotubes with cerium tungstate while preserving their tubular morphology. The XRD analyses also revealed the presence of new phases of CeO_2_ and WO_3_ on the surface of NaTiNTs as well as an increase in interlayer spacing due to the modification of these nanoparticles. XPS and STEM analyses indicated the deposition of Ce- and W-containing species on the surfaces of the nanotubes. The calculation of the Urbach energy indicates an increase for the TiNTs@CeW nanocomposite compared with the NaTiNTs, which may be related to the increase in oxygen vacancies in the NaTiNTs caused by the insertion of CeW. This complements the information obtained from the band gap and photoluminescence properties, resulting in a higher concentration of oxygen vacancies and localized electronic states, which act as emission centers and temporary charge traps, and an increase in the redshift of possible band tail and tail-to-tail transitions. These factors support the argument for modifying the NaTiNTs with cerium tungstate nanoparticles, as made evident by the discussions presented throughout this work. Additionally, the results show that the TiNTs@CeW nanocomposite is a promising material for environmental remediation, degrading 73.84% of the antibiotic under high-concentration conditions and demonstrating excellent reusability. In contrast, NaTiNTs achieved only 26.28% degradation under the same experimental conditions. These results highlight the potential of nanocomposite TiNts@CeW as effective materials in environmental remediation through their good photocatalytic performance in the degradation of the drug tetracycline. Future studies focused on mineralization pathways, intermediate identification via LC-MS, and ecotoxicological assessment will further consolidate the environmental applicability of this photocatalytic system.

## Supplementary Material



## Notes

SEM micrograph of the TiNTs@CeW sample, along with the corresponding EDS elemental maps for Na Kα, O Kα, Ti Kα, Ce Lα, and W Lα, illustrating the spatial distribution of Ce and W within the analyzed region. TEM images of the TiNTs@CeW sample after three photocatalytic reuse cycles in four different positions on the sample support, showing that there was no change in its morphology (PDF)
