# Supplementary material for: Structural, Optical, and Vibrational Properties of Cerium Tungstate–Titanate Nanocomposite for Tetracycline Degradation
Source: ACS Omega. 2026 May 21;11(21):31383–97. doi: 10.1021/acsomega.6c01535 (PMC13234892; doi:10.1021/acsomega.6c01535)
Supplement: Supplementary file 1 [file ao6c01535_si_001.pdf]

Structural, optical, and vibrational properties of cerium tungstate-titanate nanocomposite for tetracycline degradation

Emerson da Silva do Nascimento[a*], Suziete Batista Soares Gusmão[a,b], José Ferreira da Silva Júnior[c,d], Antonio Werbeson Miranda[a], Francisco das Chagas Silva Santos[a], Rodrigo Prado Feitosa[c,e], Tainara Gomes de Oliveira[c], Alexandre Silva Santos[f], Thiago de Lourenço e Vasconcelos[g], Mônica Rodrigues de Sá[h], Eduardo Padrón-Hernández[h], Bartolomeu Cruz Viana[a,c,i] and Gustavo Oliveira de Meira Gusmão[a,j*]

[a] *Graduate Program in Physics, Federal University of Piauí, Teresina, PI, CEP, 64049-550, Brazil*

[b] *Codó Science Center, Federal University of Maranhão, MA, CEP 65400-000, Brazil*

[c] *Graduate Program in Materials Science and Engineering, Federal University of Piauí, Teresina, PI, CEP, 64049-550, Brazil*

[d] *Federal Institute of Piauí, Campus Oeiras, Oeiras do Piauí, PI, CEP, 64500-000, Brazil*

[e] *Department of Analytical Chemistry, Faculty of Pharmacy, University of Sevilla, ES, Sevilla, 41012, Spain*

[f] *Optical Spectroscopy Laboratory, Instituto de Física, Universidade de Brasília, Brasília-DF, 70910-900, Brazil*

[g] *Instituto Nacional de Metrologia, Qualidade e Tecnologia (INMETRO), Duque de Caxias, RJ 25250-020, Brazil*

[h] *Department of Physics, University of Pernambuco, Av. Prof. Luiz Freire s/n, Recife-PE, CEP 50740-540, Brazil*

[i] *Center for Open Distance Education - CEAD, Federal University of Piauí, CEP, 64049-550, Brazil*

[j] *Department of Physics, State University of Piauí, Teresina, PI, CEP, 64002-150, Brazil.*

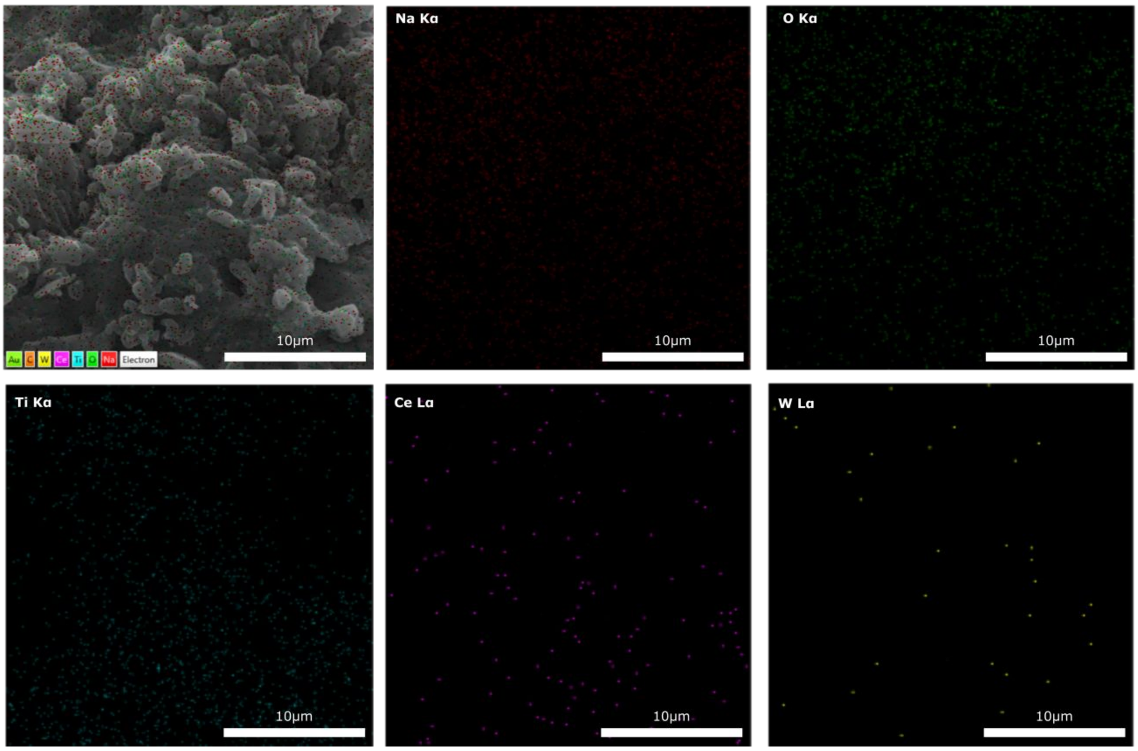

Figure SI01- SEM micrograph of the TiNTs@CeW sample and the corresponding elemental maps obtained by EDS for Na Kα, O Kα, Ti Kα, Ce Lα, and W Lα. The maps show the spatial distribution of Ce and W in the analyzed region.

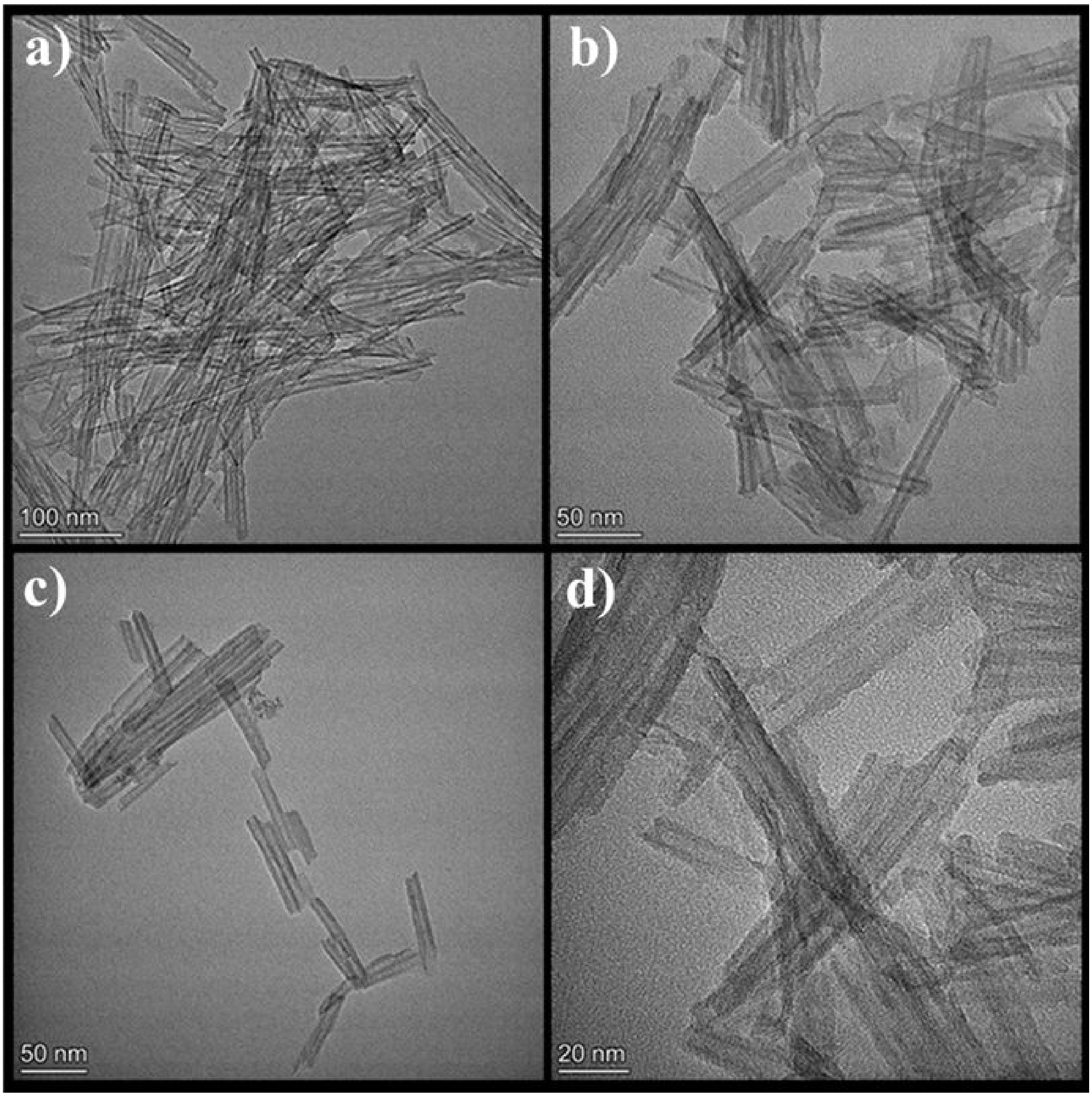

Figure SI02(a-d)- TEM image of the TiNTs@CeW sample after three photocatalytic reuse cycles in four different positions on the sample support, showing that there was no change in its morphology.
